# Supplementary material for: Length of Nutritional Transition Associates Negatively with Postnatal Growth in Very Low Birthweight Infants
Source: Nutrients. 2021 Nov 6;13(11):3961. doi: 10.3390/nu13113961 (PMC8622897; doi:10.3390/nu13113961)
Supplement: Supplementary file 1 [file nutrients-13-03961-s001.zip › nutrients-1426353-Table S1.pdf]

Table S1. Clinical characteristics of girls and boys according to the transition phase length quartiles.

	Girls (n=117)				Boys (n=131)			
	**Short Transition ad 7 d** (n=31)	**Intermediate Transition 8–12 d** (n=64)	**Long Transition over 12 d** (n=22)	p-value	**Short Transition ad 7 d** (n=23)	**Intermediate Transition 8–12 d** (n=68)	**Long Transition over 12 d** (n=40)	p-value
**Clinical characteristics, n (%)**								
Bronchopulmonary dysplasia	8 (25.8)	33 (51.6)	11 (50)	0.05[a]	16 (69.6)	37 (54.4)	22 (55.0)	0.42 [a]
Respiratory distress syndrome	20 (64.5)	44 (68.8)	15 (68.2)	0.9[a]	17 (73.9)	46 (67.6)	26 (65.0)	0.9[a]
Patent ductus arteriosus	19 (61.3)	46 (71.9)	15 (68.3)	0.6[a]	14 (60.9)	46 (67.6)	25 (62.5)	0.8[a]
Sepsis	5 (16.1)	12 (18.8)	6 (27.3)	0.6[a]	8 (34.8)	13 (19.1)	15 (37.5)	0.6[a]
Severe IVH (grade III or IV)	4 (12.9)	8 (12.5)	1 (4.5)	0.7[b]	4 (17.4)	9 (13.2)	1 (2.5)	0.08[b]
Discharged before 42 weeks of PMA	24 (87.1)	46 (71.9)	15 (68.2)	0.2[a]	17 (73.9)	51 (75.0)	28 (70.0)	0.7[a]

IVH = intraventricular hemorrhage, PMA = postmenstrual age, a = chi-squared test, b=Fisher's exact test.
