# Supplementary figures and images for: Length of Nutritional Transition Associates Negatively with Postnatal Growth in Very Low Birthweight Infants
Source: Nutrients. 2021 Nov 6;13(11):3961. doi: 10.3390/nu13113961 (PMC8622897; doi:10.3390/nu13113961)

— Enteral — Parenteral — Total

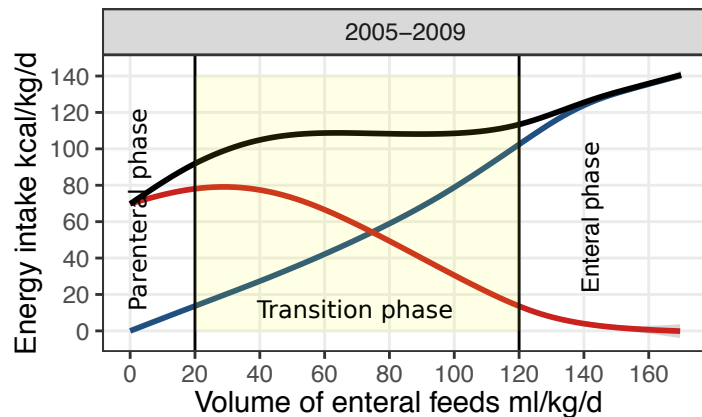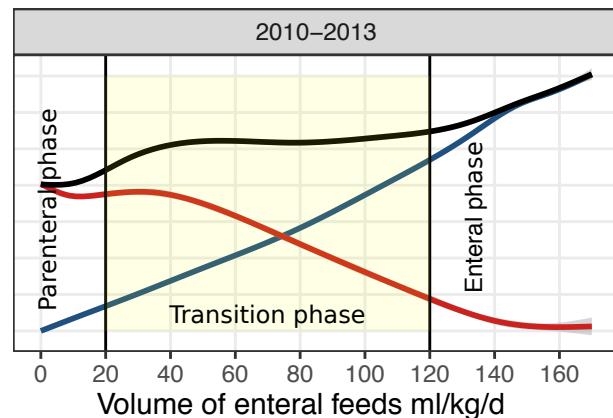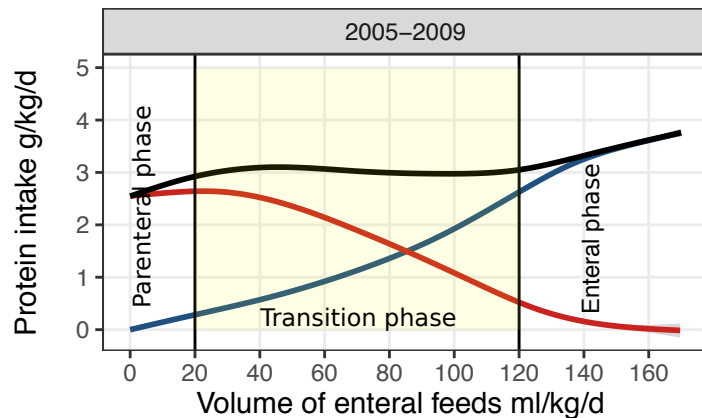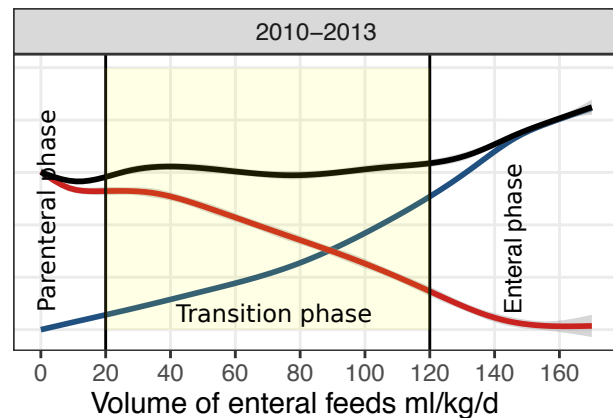

Supplement: Supplementary file 1 [file nutrients-13-03961-s001.zip › nutrients-1426353-Figure S1.pdf]
